# Identification of hub genes related to silicone-induced immune response in rats

**DOI:** 10.18632/oncotarget.21546

**Published:** 2017-10-06

**Authors:** Xiaolu Huang, Yiwen Zhou, Wenhui Liu, Haizhou Li, Xiao Liang, Rui Jin, Hengyu Du, Jizhou He, Bangda Chai, Ran Duan, Qingfeng Li

**Affiliations:** ^1^ Department of Plastic and Reconstructive Surgery, Shanghai Ninth People’s Hospital, Shanghai Jiao Tong University School of Medicine, Shanghai 200011, P.R.China

**Keywords:** silicone implant, autoimmunity, microarray, WGCNA, hub genes

## Abstract

Silicone implants are used widely in the field of plastic surgery and are used in a large population. However, their safety profile, especially the silicone-induced immune response, has been a major concern for plastic surgeons for decades. It has been hypothesized that there is a cause and effect relation between silicone and immunity, but this is controversial. The objective of the present study was to determine the hub genes and key pathways related to silicone implant–induced immune responses in a rat model. In addition to cluster and enrichment analyses, we used weighted gene co-expression network analysis (WGCNA) to examine the gene expression profiles in a systematic context. A total five genes (*Fes*, *Aif1*, *Gata3*, *Tlr6*, *Tlr2*) were identified as hub genes that are most likely related to the silicone-induced immune response, four of which (*Aif1*, *Gata3*, *Tlr6*, *Tlr2*) have been associated with autoimmunity as target genes or disease markers. The Toll-like receptor signaling pathway (*p* < 0.01, fold enrichment: 7.01) and systemic lupus erythematosus signaling pathway (*p* < 0.05, fold enrichment: 5.01), which are considered strongly associated with autoimmunity, were significantly enriched in the silicone-implanted skin samples. The results indicate that silicone implants might trigger the localized immune response, as various immune reaction genes were detected after silicone implantation. The identified five hub genes will hopefully serve as novel therapeutic targets for silicone-related complications and the associated autoimmune diseases.

## INTRODUCTION

In past decades, millions of people have been exposed to silicone under different circumstances, especially in the area of plastic and reconstructive surgery, where silicone implants are considered the most popular candidate tools for augmentation (e.g. breast implants, tissue expanders, nasal prostheses). Given the wide application and large population exposure, the safety profile of silicone implantation has become a major public health concern.

Apart from common silicone-induced complications (capsule formation and contracture), the fact that silicone implants can increase the risk of connective tissue disease or even autoimmunity has aroused great interest from both researchers and doctors. The US Food and Drug Administration (FDA) limited the use of silicone-filled breast implants in 1992 due to safety concerns [1]. Although the FDA finally reversed its decision for lack of robust evidence, the controversy around this issue has never abated. The evidence-based meta-analysis by Janowsky et al. [2] concluded that the adjusted relative risk between breast implants and connective tissue disease was 0.8 (95% confidence interval [CI]: 0.62–1.04). The authors reached this negative conclusion without including some strongly positive population-based cohort studies [3], which therefore prompted some queries [4].

On the other hand, some large-scale population-based epidemiological investigations have verified the postulated association between silicone implants and autoimmunity [5–7]. The improvement of clinical manifestations after implant removal further supports the relationship between silicone and autoimmunity [8]. Though Cohen Tervaert JW et al. has demonstrated that silicone implants could activate around inflammatory cells which contributes to an autoimmune condition [9], the underlying molecular mechanism of this development and progression has been rarely addressed.

Hence, we used dynamic microarray expression datasets and performed a primary investigation aiming to reveal the hub genes (the topmost interconnected genes that are considered to be the backbones of the co-expression networks [10, 11]) and key pathways involved in the silicone implant–induced local immune response. Comprehensive bioinformatics analyses were used to enrich datasets for Gene Ontology (GO) and pathway information to provide deeper insight into the biological process of the local immune response after silicone implantation. Furthermore, we used dynamic co-expression network construction (similar patterns of connection strengths) and gene connectivity detection to predict the hub genes most likely to contribute to the silicone-induced immune response. A total five genes (*Fes*, *Aif1*, *Gata3*, *Tlr6*, *Tlr2*) and nine pathways were identified as central participants in the silicone-induced immune response, most of which are also related to autoimmunity. These genes and pathways will hopefully serve as novel therapeutic targets for silicone-related complications and associated diseases.

## RESULTS

### Cluster analysis of significant differential genes

**Figure 1:**
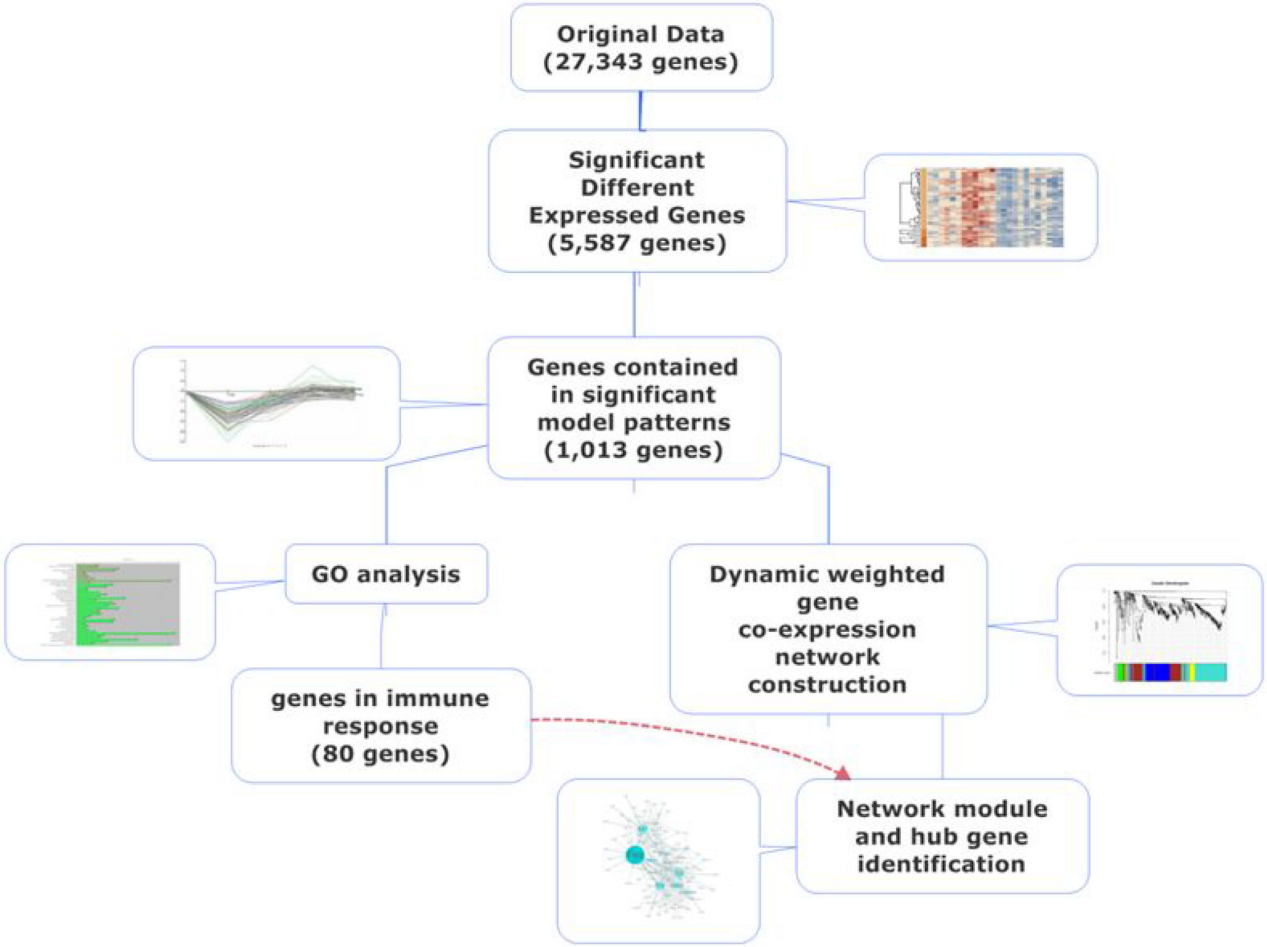
Flow chart of bioinformatics analyses of hub genes and pathways related to immune response after silicone implantation

**Figure 2:**
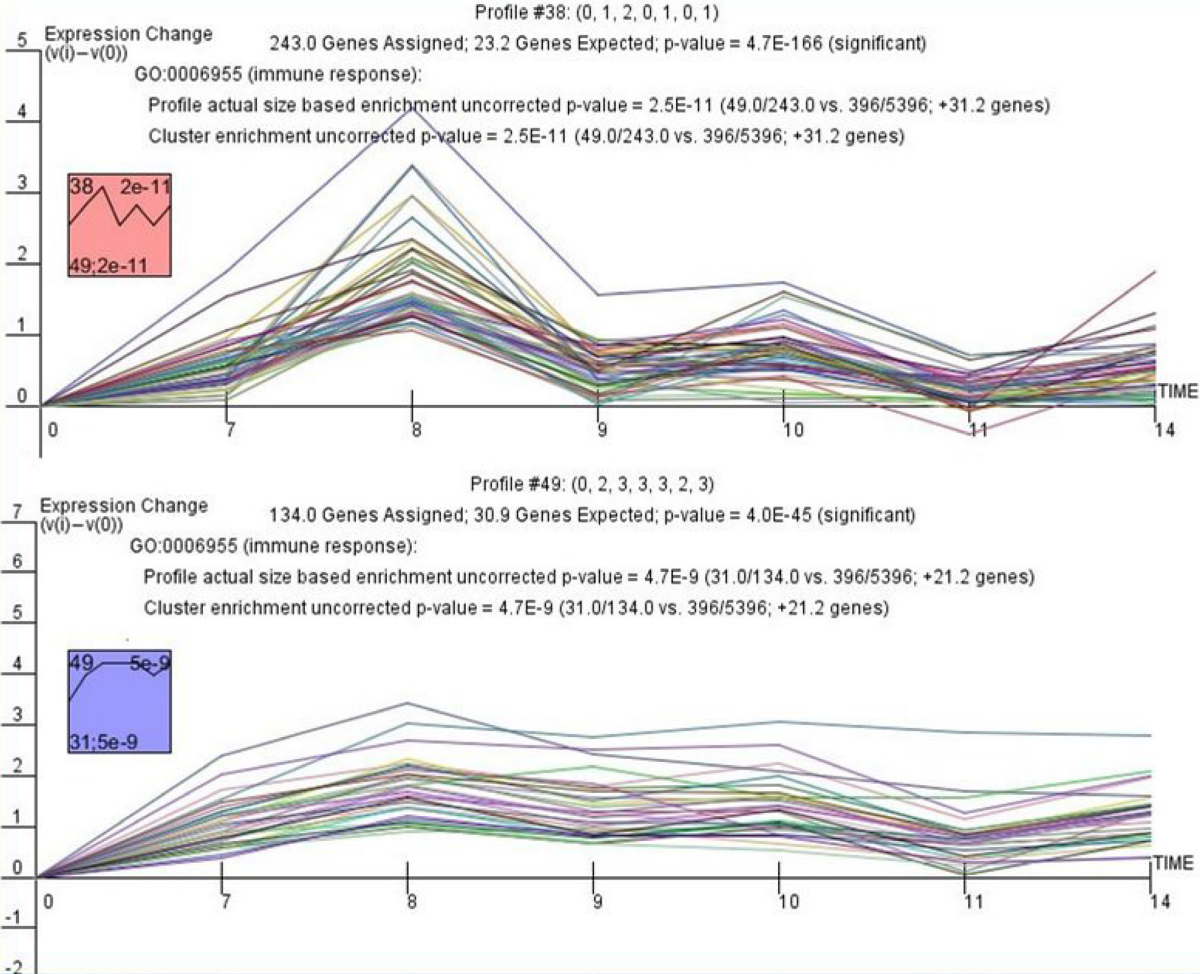
STEM cluster analysis of 80 significantly differential genes after silicone implantation Two trends (*p* < 0.001) with a statistically significant number of genes were assigned. The boxes on the left side of the figure contain detailed information on these two profiles. In the boxes, the number in the top left corner represents the profile ID, the bottom right corner shows the *p*-value, and the bottom left corner shows the gene number assigned to the profile. Details of the genes mapped to each temporal profile are in Supplementary Table 2.

A total 5,587 genes were identified as differentially expressed (*p* < 0.05), and 1,013 genes were included in significant model patterns (Figure 1), of which 117 genes were enriched in immune response (GO:0006955, *p* = 2.5E–11, Supplementary Table 1). When clusters and then profiles were ordered based on actual size and the *p*-value of gene enrichment in immune response (GO:0006955), eight profiles had a significant model pattern and five clusters were significantly enriched in the target GO term. Followed by intersection analysis, two profiles (80 genes) were statistically significant for both expression pattern and GO term enrichment (Figure 2); the expression information of these genes is detailed in Supplementary Table 2.

### GO analysis based on cluster analysis

GO analysis was used to identify a subset of the differentially expressed genes corresponding to immune response. A total 80 genes from cluster analysis were assigned to GO terms (Supplementary Tables 1, 2). After filtering using the significant criterion of corrected *p* < 0.05, we selected 24 GO terms with key functional classifications (Table 1).

**Table 1:** The summary of GO terms in significant expression patterns profiles

Category ID	Category Name	Genes Category	Genes Assigned	Genes Expected	Genes Enriched	*p-*value	Corrected *p*-value	Fold
**Profile 38**								
**GO:0006955**	immune response	396	49	17.8	31.2	2.50E-11	< 0.001	2.7
**GO:0045321**	leukocyte activation	286	35	12.9	22.1	3.60E-08	< 0.001	2.7
**GO:0009617**	response to bacterium	275	34	12.4	21.6	4.40E-08	< 0.001	2.7
**GO:0009611**	response to wounding	476	48	21.4	26.6	4.50E-08	< 0.001	2.2
**GO:0002252**	immune effector process	226	30	10.2	19.8	6.00E-08	< 0.001	2.9
**GO:0002682**	regulation of immune system process	401	42	18.1	23.9	1.30E-07	0.002	2.3
**GO:0050865**	regulation of cell activation	212	28	9.5	18.5	1.90E-07	0.002	2.9
**GO:0002237**	response to molecule of bacterial origin	230	29	10.4	18.6	3.10E-07	0.002	2.8
**GO:0032101**	regulation of response to external stimulus	248	30	11.2	18.8	4.80E-07	0.002	2.7
**GO:0046649**	lymphocyte activation	238	29	10.7	18.3	6.50E-07	0.004	2.7
**GO:0032496**	response to lipopolysaccharide	221	27	10	17	1.60E-06	0.004	2.7
**GO:0006954**	inflammatory response	271	30	12.2	17.8	3.20E-06	0.004	2.5
**GO:0002366**	leukocyte activation involved in immune response	76	14	3.4	10.6	5.50E-06	0.01	4.1
**GO:0002263**	cell activation involved in immune response	76	14	3.4	10.6	5.50E-06	0.01	4.1
**GO:0002694**	regulation of leukocyte activation	189	23	8.5	14.5	1.00E-05	0.022	2.7
**GO:0051249**	regulation of lymphocyte activation	163	21	7.3	13.7	1.10E-05	0.022	2.9
**GO:0002274**	myeloid leukocyte activation	75	13	3.4	9.6	2.40E-05	0.034	3.8
**Profile 49**								
**GO:0006955**	immune response	396	31	9.8	21.2	4.70E-09	< 0.001	3.2
**GO:0002682**	regulation of immune system process	401	28	10	18	3.50E-07	< 0.001	2.8
**GO:0006954**	inflammatory response	271	22	6.7	15.3	6.30E-07	< 0.001	3.3
**GO:0002684**	positive regulation of immune system process	261	21	6.5	14.5	1.40E-06	< 0.001	3.2
**GO:0002252**	immune effector process	226	18	5.6	12.4	9.60E-06	0.01	3.2
**GO:0002274**	myeloid leukocyte activation	75	10	1.9	8.1	1.30E-05	0.016	5.4
**GO:0002696**	positive regulation of leukocyte activation	128	13	3.2	9.8	1.40E-05	0.018	4.1
**GO:0002675**	positive regulation of acute inflammatory response	23	6	0.6	5.4	1.50E-05	0.024	10.5
**GO:0030097**	hemopoiesis	260	19	6.5	12.5	1.80E-05	0.028	2.9
**GO:0002823**	negative regulation of adaptive immune response based on somatic recombination of immune receptors built from immunoglobulin superfamily domains	15	5	0.4	4.6	2.20E-05	0.04	13.4
**GO:0002820**	negative regulation of adaptive immune response	16	5	0.4	4.6	3.10E-05	0.046	12.6
**GO:0002697**	regulation of immune effector process	119	12	3	9	3.30E-05	0.05	4.1

An interaction network of significant GO terms was assembled into a GO map using ClueGO to depict the relationship among prominent functional categories (Supplementary Figure 1). Based on the GO map, we were able to directly and systematically find the subordinate relationship between GO terms. Comparison of the comprehensive GO analysis and cluster GO analysis suggested that immune response, leukocyte activation, lymphocyte activation and immune effector process, and signaling processes were the most prominent functions after silicone implantation.

### Pathway analysis

**Figure 3:**
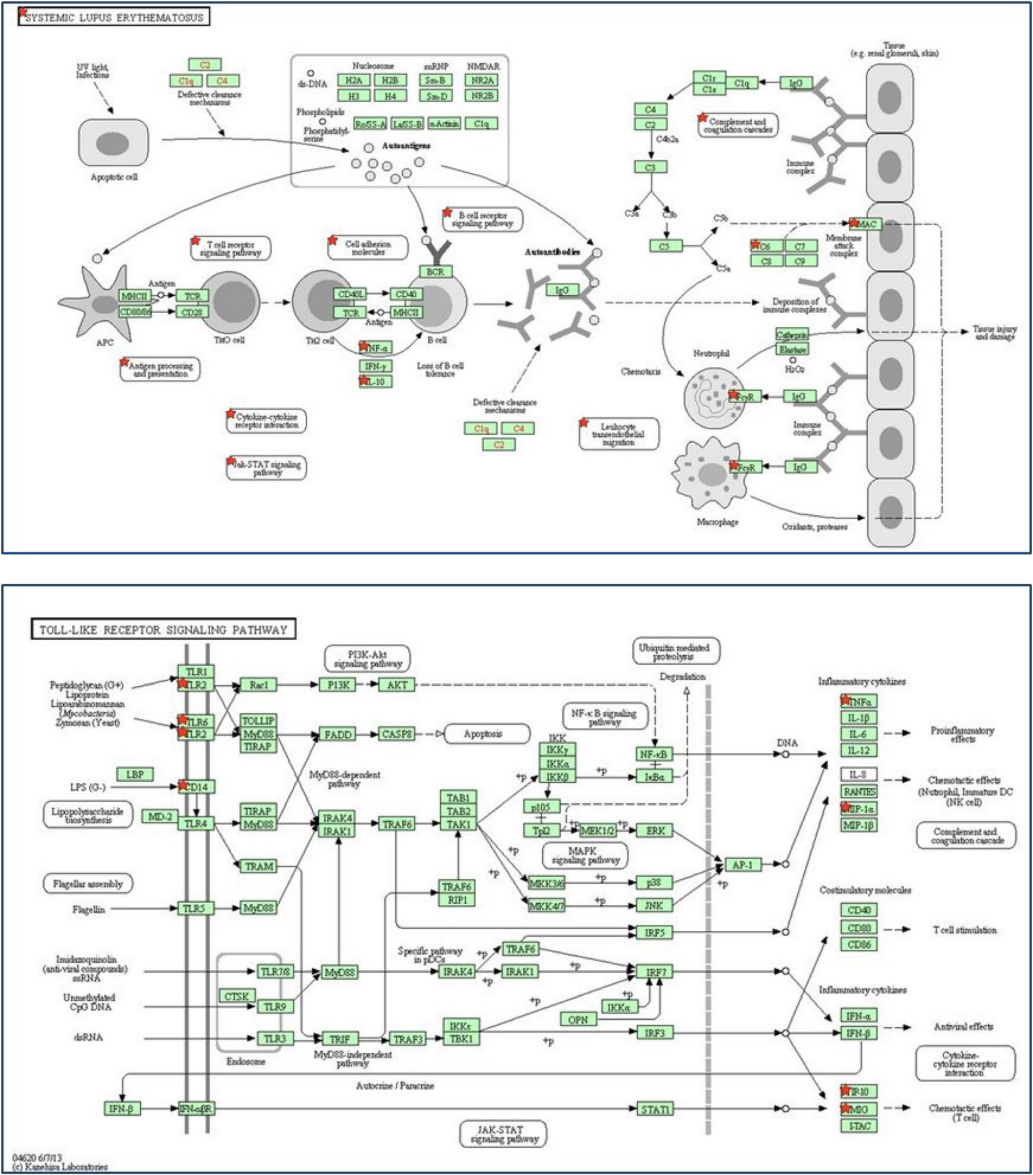
Identification of two autoimmunity-related signaling pathways Top: SLE signaling pathway; bottom: TLR signaling pathway. Red stars indicate the significantly expressed genes in the pathways.

We used DAVID software based on the KEGG pathway map to investigate key pathways linked to the 80 genes. Our analysis yielded nine statistically significant pathways (Table 2) involving hematopoietic cell lineage, Toll-like receptor (TLR) signaling, and NOD-like receptor signaling. The association between autoimmunity and silicone implantation was highlighted by the identification of the systemic lupus erythematosus (SLE) signaling pathway (Figure 3, top) and TLR signaling pathway (Figure 3, bottom).

**Table 2:** Summary of statistically significant key pathways

Term	Genes	Count	%	*P*-Value	Fold Enrichment	FDR
Cytokine-cytokine receptor interaction	CSF3, CCL3, TNF, CCL2, CSF1, CXCL2, CXCL9, TNFSF13, PF4, IL7R, TNFSF18, CCL4, IL10, CXCL10, OSM, CSF1R	16	1.714898	1.60E-09	7.322744	1.73E-06
Hematopoietic cell lineage	CSF3, CD38, CD55, CD37, TNF, CSF1, FCGR1A, IL7R, CD14, CSF1R	10	1.071811	1.21E-07	11.55914	1.31E-04
Chemokine signaling pathway	CXCL1, CCL3, CCL2, CXCL2, CXCL9, CCL9, JAK2, PF4, CCL4, CCL7, CCL6, CXCL10	12	1.286174	1.76E-06	6.327108	0.001906
Toll-like receptor signaling pathway	CCL3, TNF, TLR2, CXCL9, TLR6, CD14, CXCL10	7	0.750268	4.02E-04	7.012545	0.433237
NOD-like receptor signaling pathway	CXCL1, TNF, CCL2, CXCL2, CCL7	5	0.535906	0.004465	7.271072	4.721757
Systemic lupus erythematosus	TNF, FCGR2B, C6, FCGR1A, IL10	5	0.535906	0.01632	5.008961	16.29149
Jak-STAT signaling pathway	OSM, CSF3, STAT5A, JAK2, IL7R, IL10	6	0.643087	0.017261	3.891854	17.15306
Natural killer cell mediated cytotoxicity	ITGAL, TNF, FCGR2B, FCER1G, TYROBP	5	0.535906	0.023113	4.508065	22.33229
Asthma	TNF, FCER1G, IL10	3	0.321543	0.025582	11.76017	24.4271
Fc gamma R-mediated phagocytosis	PTPRC, GAB2, FCGR2B, FCGR1A	4	0.428725	0.070663	4.09824	54.70701
Cell adhesion molecules (CAMs)	ALCAM, ITGAL, PTPRC, CD274, SPN	5	0.535906	0.077219	3.04599	58.04327
Intestinal immune network for IgA production	TNFSF13, IL10, TGFB1	3	0.321543	0.086023	6.010753	62.17279
Cytosolic DNA-sensing pathway	IL33, CCL4, CXCL10	3	0.321543	0.089322	5.880084	63.62241

### WGCNA and hub gene detection

We selected 1,013 genes for further analysis by constructing a weighted gene co-expression network. First, we identified six network modules, which are illustrated in the dendrogram (Supplementary Figure 2). In network terminology, modules are groups of genes with similar patterns of connection strengths with all other genes in the network and they usually share similar functions [12].

**Figure 4:**
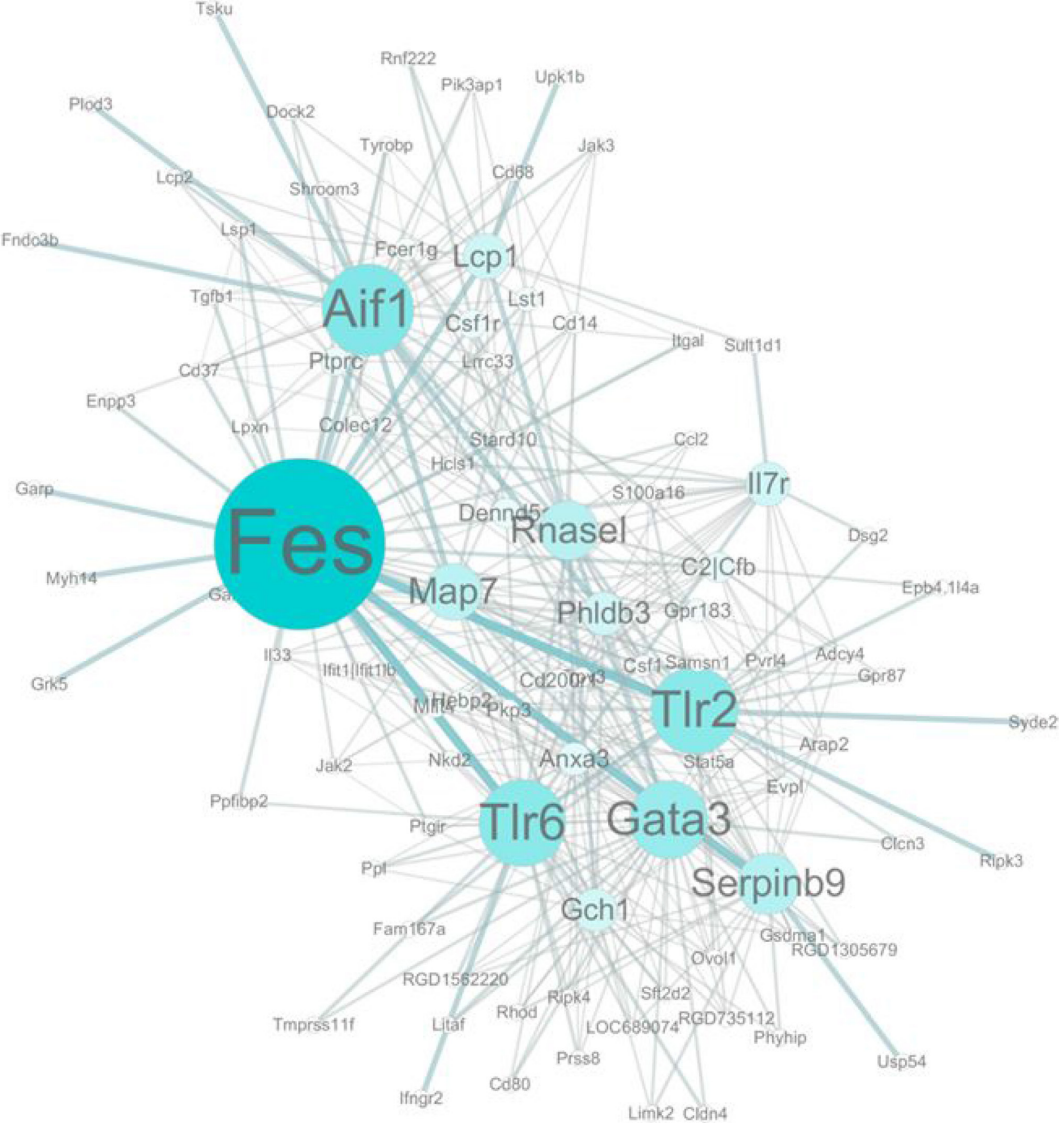
Gene co-expression network Genes contained in significant GO terms were analyzed and identified by the gene co-expression network using the k-core algorithm. Nodes represent genes; edges indicate the interaction between the genes. The area of each node represents the k-core value within the module, and the edge correlates with the capacity for modulating adjacent genes. Genes with higher k-core values are more centralized in the network and have a stronger capacity for modulating adjacent genes.

**Figure 5:**
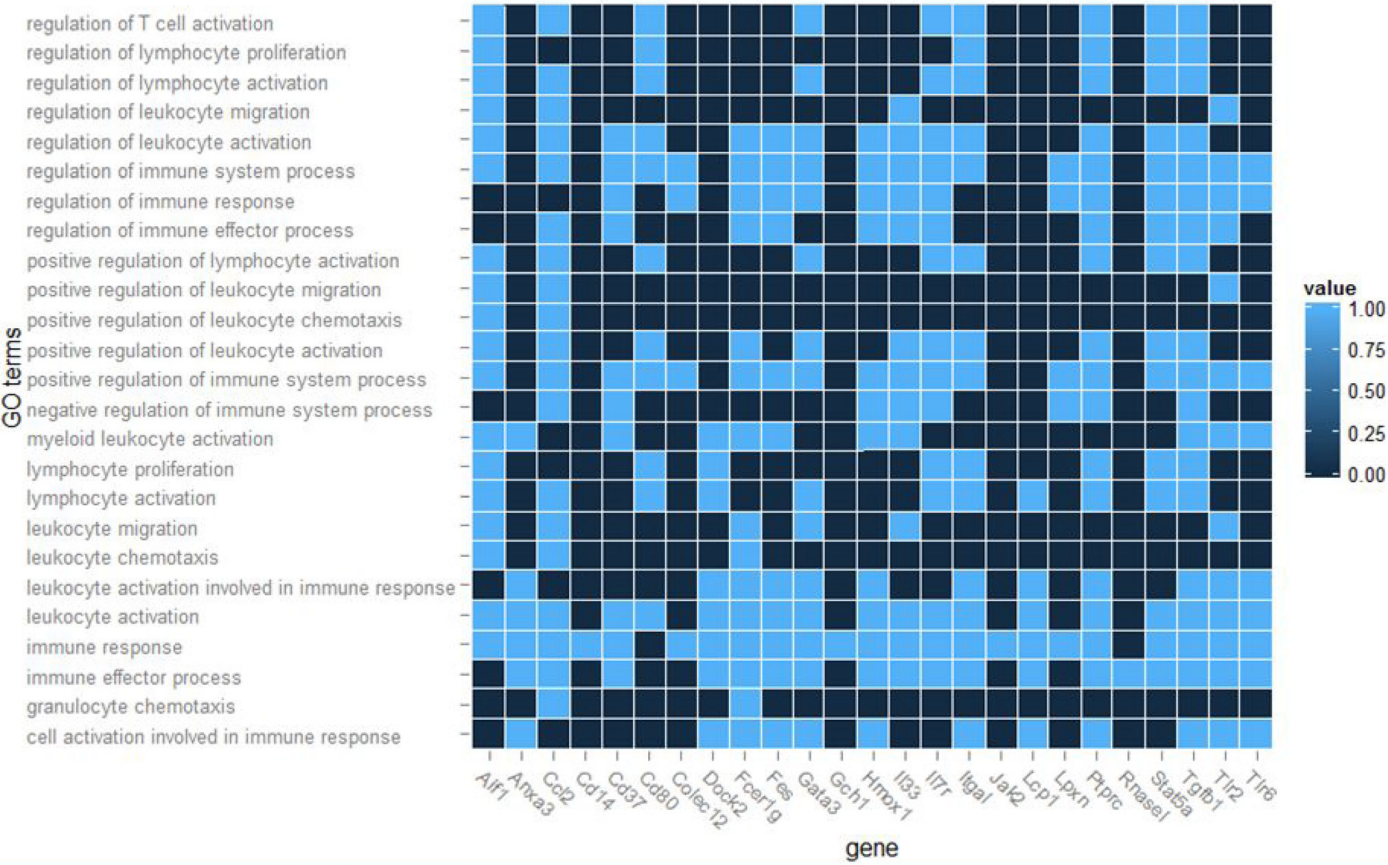
Heat map of distribution of top 25 genes in 25 significant GO terms The bar on the right indicates if the gene is a participat in the GO term (light blue, yes; dark blue, no).

After module detection, the connection strength between two genes was determined using soft thresholding of the Pearson correlation matrix [12]. Figure 4 demonstrates the co-expression network of the 80 selected genes, which reflects the correlations between the genes. Each node describes a given gene, and the relationship between a pair of genes is represented with an edge. Further, the area of the node represents its k-core value within the module, and the edge correlates with the capacity for modulating adjacent genes. Genes with higher k-core values are more centralized in the network and have a stronger capacity for modulating adjacent genes. Consequently, we identified five genes with the highest k-core value in the network as hub genes in the immune response after silicone implantation, namely, *Fes*, *Aif1*, *Tlr6*, *Tlr2*, and *Gata3*. Twenty-five genes with the highest k-core values were subsequently assigned to significant GO terms to investigate the distribution of their participation; Figure 5 depicts the heatmap.

### Real-time PCR validation

The relative expression levels of the five differentially expressed genes (*Fes*, *Aif1*, *Tlr6*, *Tlr2*, *Gata3*) were assayed. The results of the microarray assay and RT-PCR were consistent (Supplementary Figure 3).

## DISCUSSION

The present study yields information on the hub genes and key pathways related to the silicone implant–induced immune response as determined using integral bioinformatics methods. We aimed to better understand and characterize local inflammatory and immunologic reactions caused by silicone implants as groundwork to clarify whether this local immunologic reaction can lead to subsequent systemic immune reactions, and even autoimmune diseases. Importantly, by utilizing novel bioinformatics techniques, we bridge the gap between individual genes and systematic biology. The detected hub genes and key pathways are expected to be future therapeutic targets for silicone implant–induced complications and diseases.

Five genes with the highest k-core values were identified as hub genes related to the silicone-induced immune response. Strikingly, most of these hub genes (*Tlr2*, *Tlr6*, *Aif1*, *Gata3*) have also been reported to be crucial in autoimmunity development. They not only give rise to the localized silicone-induced immune response, but also function as central players in autoimmune disease development [13–18].

As important components of foreign entity recognition localized on antigen-presenting cells, TLRs are the key signaling molecules in innate immune activation [19]. The downstream activated signaling pathway, termed the TLR signaling pathway (Figure 3, bottom), is the key element of not only adjuvant-induced immune response [5], but also the therapy target in autoimmune disease [19]. In the present study, we found that *Tlr2* dimerized with *Tlr6* are the major receptors involved in silica surface antigen recognition. Various studies have demonstrated that abnormal and constant activation of TLRs and the TLR signaling pathway can result in sterile inflammation or autoimmunity [20, 21] and the subsequent development of a syndrome termed autoimmune syndrome induced by adjuvants (ASIA) [5, 22]. Considering the suggested linkage between autoimmunity and the silicone-induced immune response, novel material should be employed for implantation in the future to avoid activating the *Tlr2/6* signaling pathway to circumvent the common adverse effects stemming from the costly whole-body immune response to antigens.

Allograft inflammatory factor-1 (*Aif1*), the hub gene with the second highest k-core value, is expressed predominantly by activated monocytes. An increasing number of studies suggest that *Aif1* may play a critical role in the immune response to allo- or auto-antigens and inflammatory responses [13, 23–24], and its expression level parallels the autoimmune disease stage [25]. Of interest is that, in a rat model with silicone implants, Eltze et al. found a significant correlation between *Aif1*-positive macrophages and capsule thickness, which indicates that *Aif1* might be a novel marker of silicone-induced chronic immune response monitoring [15]. However, the molecular regulatory mechanism of *Aif1* expression and function is unknown. *Aif1*-1 knockout mice and modern biological technology are important for better understanding of *Aif1* immune regulatory biology.

The hub gene with the highest k-core value was feline sarcoma oncogene (*Fes*). However, it is rarely investigated in immunology and little information is currently available to derive preliminary understanding of its function and role in immune-regulation. Further study is necessary to identify specific cell lines that predominantly express *Fes* after silicone implantation and that demonstrate the exact function of *Fes* when interacting with silicones.

The imbalance of T helper cell (Th1/Th2) differentiation is considered a major pathogenesis step in autoimmune diseases [14, 26, 27]. Interestingly, three of the five hub genes (*Gata3*, *Aif1*, *Tlr2*) function as agonists for Th1 differentiation. *Gata3* (GATA Binding Protein 3) plays an indispensable role in Th2 differentiation [28], *and* its overexpression has been considered a therapeutic target in autoimmune disease [14]. Our data show that *Gata3* had a tendency to be significantly decreased after silicone implantation (Supplementary Table 1, Supplementary Figure 3), indicating imbalanced T helper cell differentiation that may give rise to Th1 autoimmune disease. Additionally, *Aif1* regulates Th1 inflammatory responses by augmenting the production of specific cytokines [29], and its expression is increased in Th1-type disease [30, 31]. The characteristic of *Tlr2* in Th1 differentiation is well-described. *Tlr2* is a specific activator of Th1 function and its involvement is implied in Th1-mediated responses [32]. The co-expression of these three genes following silicone implantation suggested abnormally increased differentiation towards Th1 and Th1/Th2 imbalance. However, little is known about how silicone affects T helper cell differentiation, and our findings might provide clues to the establishment of a novel pathway model for mimicking silicone-induced autoimmunity.

Further investigation will mainly focus on whether interfering with the expression of these hub genes would significantly exacerbate or alleviate the immune response after silicone implantation. Additionally, these data will be further validated and replicated with silicone implants in human samples. The shared target genes and common pathways between the silicone-induced immune response and population-based connective tissue disease microarray data (from the Gene Expression Omnibus [GEO] database) will also be compared and analyzed for further study.

Our study has limitations: the time points span a relatively short period compared to that in human patients, who bear silicone implants for years. However, this setting is based on the principle of co-expression network construction, which mainly depends on relative expression changes between two genes from different samples, rather than the expression level of one single gene [33]. Consequently, the expression level profiles of the single genes we report are of lower reference value than the information on the hub genes, which can only represent the acute immune response triggered by silicone implants. Therefore, the expression results at single gene–level should be interpreted with care (Supplementary Tables 1, 2).

With the integral bioinformatics approach, especially WGCNA, we were able to identify the hub genes of the silicone-induced immune response, and thereby infer its potential relationship with autoimmunity, which is controversial and has perplexed epidemiologists for decades. We hope that the information presented here will prompt not only scientists to develop interference medicines, but also encourage manufacturers to improve silicone surface antigens to elude immune recognition for the purpose of alleviating the suffering of patients with silicone-caused complications and associated diseases.

## MATERIALS AND METHODS

### Animals and silicone implantation

Ethics statement: All animal studies complied with current ethical considerations in Directive 2010/63/EU. We used male specific pathogen–free Sprague-Dawley (SD) rats (8 weeks old; male, average body weight, 250–300 g).

The rats were randomly allocated to silicone implantation (*n* = 18, intervention) or sham (*n* = 3, control) groups. in the intervention group were randomly assigned to six time points (*n* = 3 rats per time point): 7, 8, 9, 10, 11, and 14 days. The tissue expander and implantation procedures were described in previous studies [34, 35]. Briefly, the rats were implanted with 10-ml silicone tissue expanders (silicone MED4735, Shanghai Xinsheng Biomedical Co. Ltd, Shanghai, China; http://www.xinsheng-sh.net/) subcutaneously on the dorsal side (Supplementary Figure 4), and 30 ml saline was injected through the pot to mimic the mechanical stretching after silicone implantation. Rats in the control group underwent a sham procedure without implanting the silicone expanders. At each time point, 0.5 cm × 0.5 cm full-thickness skin specimens (including the capsule) from the implanted skin area were collected. In the control group, 0.5 cm × 0.5 cm full-thickness skin specimens from the dorsal midline were collected at day 7. The animals were euthanized immediately after sample collection. The experiments were performed in accordance with the National Experimentation Manual.

### RNA isolation

Total RNA was extracted from the skin specimens, added to TRIzol (Invitrogen, Carlsbad, CA, USA) per the manufacturer’s protocol, and was purified using RNeasy Mini columns (Qiagen, Valencia, CA, USA). The absorbance ratio at 260/280 nm (OD 260/280) was measured to evaluate the purity of all RNA samples; RNA quality and integrity were assessed using agarose gel electrophoresis. The 28S to 18S rRNA band intensity ratio of all RNA samples was approximately 2:1; the OD 260/280 ratio was 2.0–2.1.

### Microarray experiment

RNA samples from all groups were used to detect gene expression changes at each time point. Hybridization, washing, and scanning of Affymetrix GeneChip Rat Exon 1.0 ST Arrays (Santa Clara, CA, USA) were performed according to standard Affymetrix protocols. Statistically significant gene expression was determined using two-way analysis of variance (ANOVA, *p* < 0.05) because we investigated two factors (silicone implantation and time course). All data generated in this study were minimum information about a microarray experiment (MIAME)-compliant [36].

### Bioinformatics analysis of microarray data

Comprehensive bioinformatics analysis was used to determine the hub genes related to the silicone implantation–induced immune response, and included: cluster analysis, GO analysis, pathway analysis, dynamic gene network construction, and hub gene identification (Figure 1).

### Cluster analysis

A total 5,587 significantly expressed genes were assigned to cluster analysis by Short Time-series Expression Miner (STEM) version 1.4 and according to the instructions by Ernst et al. [37]. Fifty model temporal expression patterns were used to identify the expression patterns of the significantly differential genes, and were simultaneously integrated with GO classification. Each cluster contained genes with similar expression patterns. The gene clusters were ranked by the *p*-value of significance of the observed number of genes that fit a profile beyond the expected number.

### GO analysis and pathway analysis

A total 1,013 genes had a significantly differential expression pattern (*p* < 0.001), and underwent GO analysis, which is the key functional classification of the National Center for Biotechnology Information (NCBI) [38]. All GO terms assigned to these genes were obtained, and pathway analysis was enriched using the Database for Annotation, Visualization and Integrated Discovery (DAVID) [39] web server, interrogating the Kyoto Encyclopedia of Genes and Genomes (KEGG) database, and examined simultaneously using Fisher’s exact test and the χ^2^ test for calculating the level of significance. The false discovery rate (FDR) was calculated to correct the *p*-value. Significant genes related to GO terms named “immune response” were extracted for pattern identification, and the GO term interaction network was visualized using ClueGO [40].

### Dynamic weighted gene co-expression network construction

Next, we used weighted gene co-expression network analysis (WGCNA) to integrate the genes with significant expression patterns into a higher-order, systems-level context [33]. WGCNA is designed to identify modules of densely interconnected genes by searching for genes with similar patterns of connectivity with other genes, which can be summarized as the topological overlap between genes [33]. The blockwiseModules function allowed the entire dataset of 1,013 genes to be utilized in the construction of the weighted gene co-expression network. A pair-wise correlation matrix was computed for each set of genes, and the correlations were weighted to a power of β using the power function [33].

### Network module and hub gene identification

The dynamic tree-cutting algorithm [41] was then used to identify the modules of co-expressed genes. After all blocks had been processed, a gene was reassigned to another module if it had higher connectivity to the other module, and modules with highly correlated hub genes were merged [42]. Modules, or groups of highly correlated genes, could be a result of transcriptional co-activation (gene activation or gene repression), the co-regulation of mRNA stability, or a combination thereof, resulting in a complex genetic network of closely related genes coordinately operating to accomplish a function or a group of related functions [10].

The TOM function was used to calculate the connectivity (k-core value) of every signal gene in the network; genes with higher k-core values are more centralized in the network and have stronger capacity for modulating adjacent genes. Highly connected “hub” genes are of special interest because they are the backbone of the scale-free network architecture [10], and are far more likely than non-hub genes to be essential for survival in lower organisms [43–45]. As an analogy, the interference of hub genes related to the silicone-induced immune response may lead to amelioration of the pathological process.

### Real-time PCR validation of microarray data

Real-time reverse transcription (RT)-PCR was performed to verify the differential expression of five selected genes: *Fes*, *Aif1*, *Gata3*, *Tlr6*, and *Tlr2*. Total RNA was isolated from the skin specimens of rats in the intervention group via microarray assay in three independent experiments. The RNA was reverse-transcribed using a ReverTra Ace qPCR RT Kit (TOYOBO, Osaka, Japan). Real-time RT-PCR was conducted in 10-ml reactions consisting of 5 ml SYBR Green Real-time PCR Master Mix (Applied Biosystems), 0.3 mM primers, and 1 ml template complementary DNA (cDNA). The PCR program began with initial denaturation for 5 min at 94°C, followed by 40 cycles of 30 sec at 94°C, 30 sec at 60°C or 65°C, and 30 sec at 72°C, and ended with the melting curve program. The relative changes in gene expression were calculated using the comparative threshold cycle (2^–ΔΔCt^) method; β-actin was used as the internal control gene to normalize the amount of RNA used in the PCR [46].

## CONCLUSIONS

The present study results indicate that the silicone-induced immune response is related to various immune reaction genes and pathways. Five hub genes and nine signaling pathways were identified as central participants in the silicone-induced immune response, most of which are also related to autoimmunity. These genes and pathways will hopefully serve as novel therapeutic targets for silicone-related complications and associated diseases.

## SUPPLEMENTARY MATERIALS FIGURES AND TABLES







## Abbreviations

*Fes*: Feline sarcoma oncogene
*Aif1*: Allograft inflammatory factor-1
*Tlr6*: Toll-like receptor 6
*Tlr2*: Toll-like receptor 2
*Gata3*: GATA Binding Protein 3
ASIA: Autoimmune syndrome induced by adjuvants
DAVID: Database for Annotation, Visualization and Integrated Discovery
FDR: False discovery rate
GEO: Gene Expression Omnibus
GO: Gene ontology
KEGG: Kyoto Encyclopedia of Genes and Genomes
MIAME: Minimum information about a microarray experiment
NCBI: National Center for Biotechnology Information
SLE: Systemic lupus erythematosus
STEM: Short Time-series Expression Miner
TLR: Toll-like receptor
WGCNA: Weighted gene co-expression network analysis
