# Supplementary material for: Identification of hub genes related to silicone-induced immune response in rats
Source: Oncotarget. 2017 Oct 6;8(59):99772–83. doi: 10.18632/oncotarget.21546 (PMC5725130; doi:10.18632/oncotarget.21546)
Supplement: Supplementary file 1 [file oncotarget-08-99772-s001.pdf]

Identification of hub genes related to silicone-induced immune response in rats

SUPPLEMENTARY MATERIALS

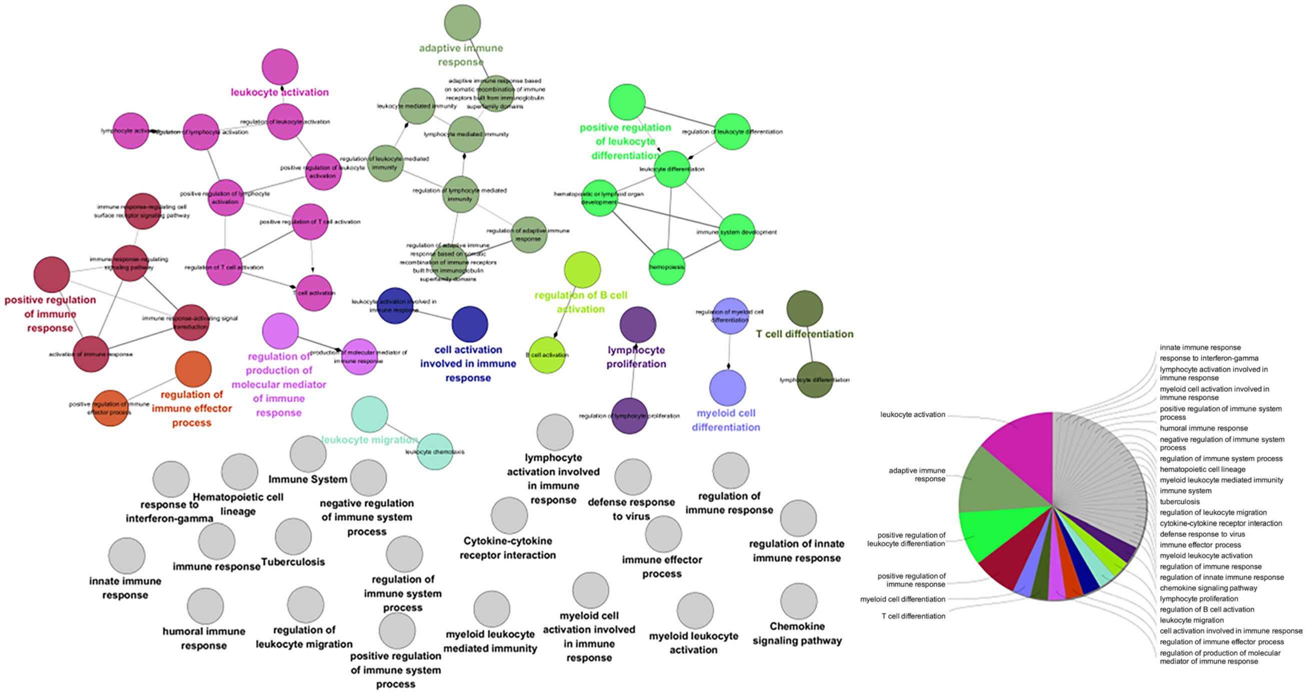

Supplementary Figure 1: The interaction network of significant GO terms. Each node represents one GO term; the edge between two nodes represents the subordinate relationship between GO terms, and the groups of a GO term are illustrated by color; the pie chart shows an overview of the clusters.

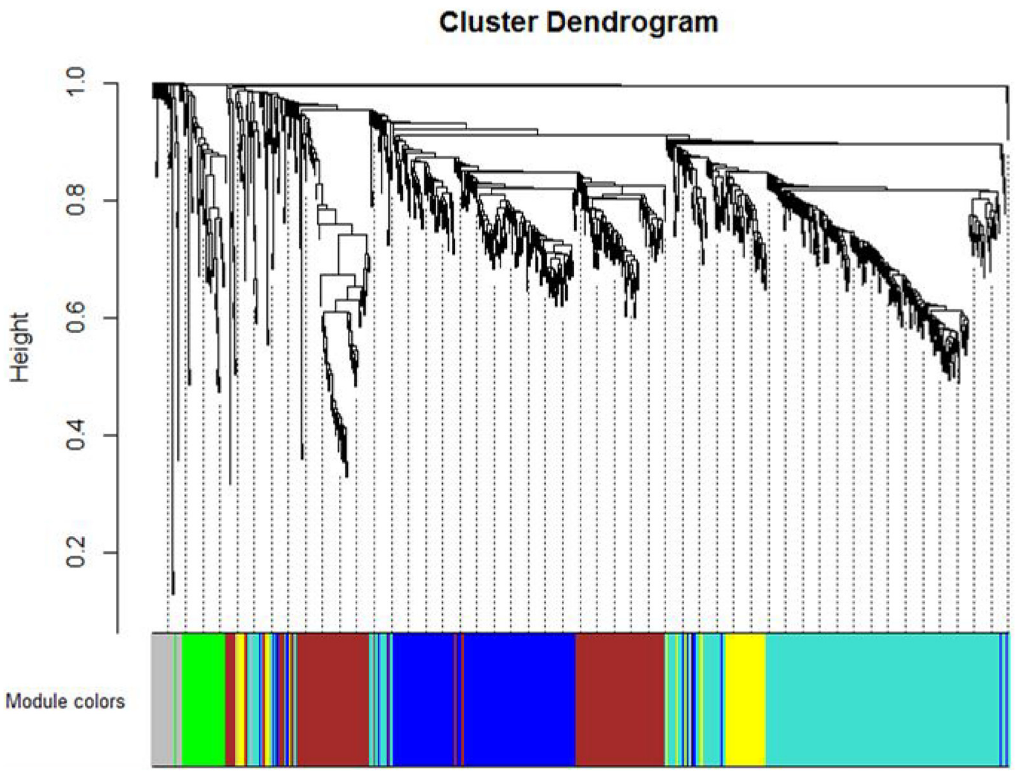

Supplementary Figure 2: Cluster dendrogram of the significantly expressed genes during the immune response after silicone implantation, with dissimilarity based on topological overlap, together with assigned module colors. A total six modules were detected.

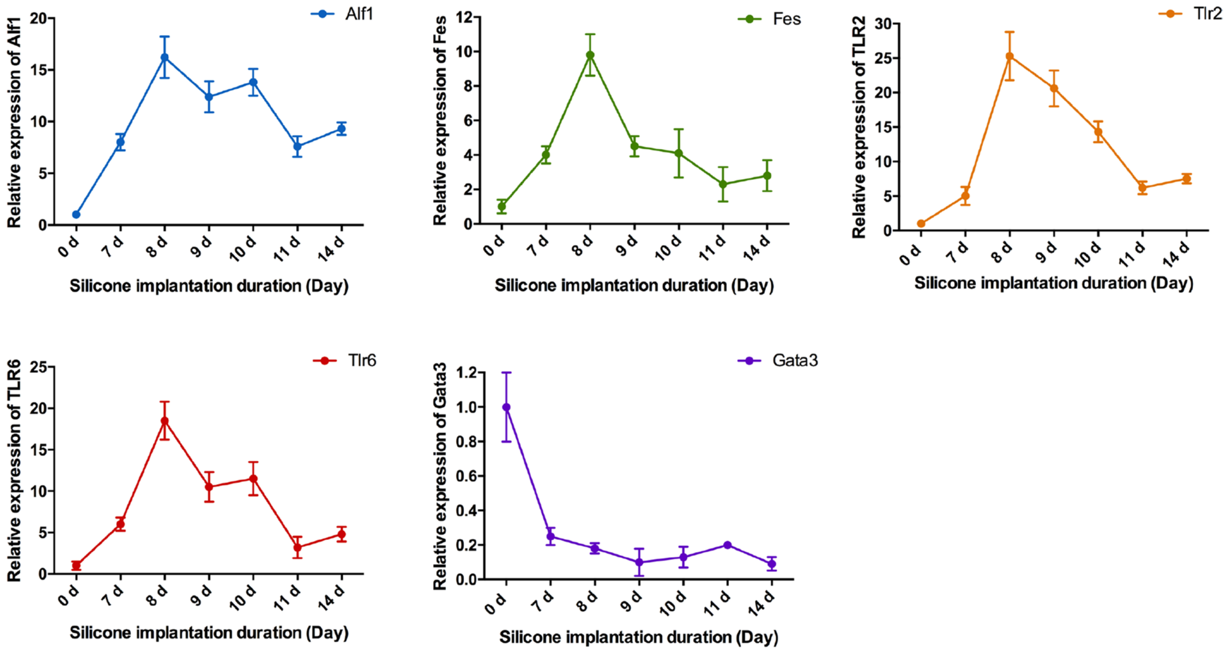

Supplementary Figure 3: Validation of microarray results of most central genes which take participant in immune response after silicone implantation. The expression fold changes of five genes of high k-core value after silicone implantation compared with control group (error bar represents SEM). The relative changes in gene expression were calculated by the $2^{-\Delta\Delta Ct}$ method, with β-actin serving as an internal control gene. The x-axis represents the silicone implantation duration, and the control group which underwent sham surgery (without silicone implantation) was regarded as 0d.

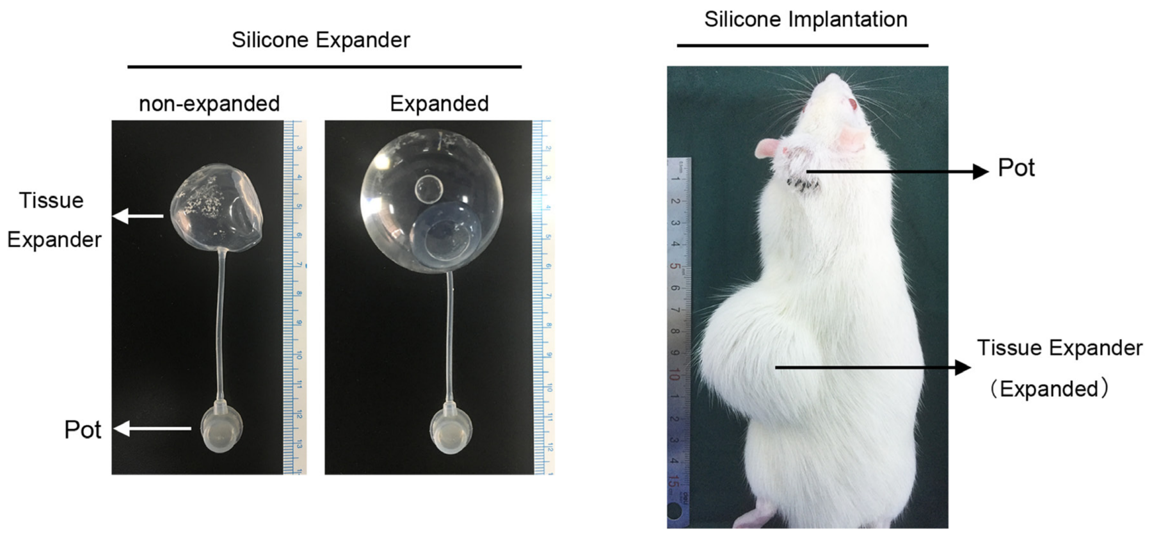

Supplementary Figure 4: Figure illustrating the silicone expander and the rat model. (Left) Showing the implanted silicone expander, and the saline was injected through the pot. (Right) Rat model with silicone expander implantation.

Supplementary Table 1: The 117 genes enriched in the immune response in the significant model patterns. See Supplementary_Table_1

Supplementary Table 2: Expression information of two statistically significant profiles (80 genes) in terms of both expression pattern and GO term enrichment. See Supplementary_Table_2
