# Supplementary material for: Identification of hub genes related to silicone-induced immune response in rats
Source: Oncotarget. 2017 Oct 6;8(59):99772–83. doi: 10.18632/oncotarget.21546 (PMC5725130; doi:10.18632/oncotarget.21546)
Supplement: Supplementary file 2 [file oncotarget-08-99772-s002.docx]

Supplementary Table 1: The 117 genes enriched in the immune response in the significant model patterns

| genesymbol | spot_id | Profile | 0 | 7 | 8 | 9 | 10 | 11 | 14 |
| --- | --- | --- | --- | --- | --- | --- | --- | --- | --- |
| RAET1C | 6 | 1 | 0 | -0.55 | -1.22 | -0.89 | -0.03 | -0.66 | -0.7 |
| ENPP3 | 35 | 38 | 0 | 0.72 | 1.58 | 0.46 | 1.34 | 0.18 | 0.7 |
| MILL1 | 139 | 20 | 0 | -0.46 | -1.79 | -0.47 | -0.41 | -0.2 | -0.54 |
| TGFB1 | 169 | 38 | 0 | 0.67 | 1.17 | 0.47 | 0.9 | 0.43 | 0.75 |
| TYROBP | 193 | 49 | 0 | 1.1 | 1.92 | 1.57 | 1.51 | 0.77 | 1.39 |
| GAB2 | 316 | 49 | 0 | 0.78 | 1.36 | 0.89 | 1.01 | 0.75 | 0.96 |
| ITGAL | 422 | 38 | 0 | 0.63 | 1.54 | 0.51 | 1.28 | 0.48 | 0.64 |
| IFITM1 | 464 | 49 | 0 | 1.72 | 2.25 | 1.79 | 2.24 | 1.15 | 1.96 |
| UNC93B1 | 484 | 49 | 0 | 0.98 | 1.6 | 1.28 | 1.59 | 0.64 | 1.41 |
| LPXN | 544 | 49 | 0 | 0.55 | 1.4 | 0.77 | 1.11 | 0.34 | 0.8 |
| JAK2 | 580 | 38 | 0 | 0.74 | 2.05 | 0.93 | 0.82 | 0.24 | 0.59 |
| CD274 | 581 | 38 | 0 | 0.53 | 1.86 | 0.56 | 0.57 | 0.38 | 0.6 |
| IL33 | 583 | 49 | 0 | 0.9 | 2.03 | 1.3 | 1.3 | 0.1 | 1.3 |
| NFKB2 | 625 | 38 | 0 | 0.39 | 1.44 | 0.73 | 0.77 | 0.37 | 0.3 |
| STX11 | 667 | 38 | 0 | 0.3 | 2.02 | 0.69 | 0.56 | 0.25 | 0.36 |
| VNN1 | 688 | 40 | 0 | 0.55 | 0.88 | 0.35 | -0.15 | 0.62 | 1.15 |
| FCGRT | 884 | 38 | 0 | 0.85 | 1.16 | 0.58 | 0.62 | 0.25 | 0.75 |
| CD37 | 888 | 49 | 0 | 0.72 | 1.77 | 1.18 | 1.43 | 0.79 | 1.31 |
| FES | 958 | 38 | 0 | 0.61 | 1.46 | 0.83 | 0.75 | 0.21 | 0.48 |
| SPN | 1093 | 49 | 0 | 0.56 | 0.91 | 0.95 | 0.92 | 0.64 | 1.24 |
| ADAM8 | 1119 | 41 | 0 | 0.58 | 0.85 | 1.28 | 1.27 | 0.75 | 0.23 |
| IFIT1 | 1249 | 38 | 0 | 0.44 | 3.38 | 0.72 | 1.11 | 0.13 | 0.25 |
| PIK3AP1 | 1269 | 38 | 0 | 0.32 | 1.26 | 0.57 | 0.8 | 0.23 | 0.52 |
| ADORA2B | 1427 | 38 | 0 | 0.94 | 2.95 | 0.75 | 0.81 | 0.05 | 0.4 |
| NOS2 | 1511 | 38 | 0 | 0.56 | 2.64 | 0.47 | 0.53 | 0.05 | 0.2 |
| CCL2 | 1523 | 49 | 0 | 2.38 | 3.43 | 2.42 | 2.09 | 1.68 | 1.6 |
| CCL7 | 1524 | 38 | 0 | 1.89 | 4.19 | 1.56 | 1.74 | 0.7 | 0.87 |
| CCL4 | 1534 | 38 | 0 | 0.13 | 2.19 | 0.46 | 0.36 | 0.09 | 0.35 |
| CSF3 | 1582 | 38 | 0 | 0.08 | 1.41 | 0.08 | 0.1 | 0.09 | 0.08 |
| STAT5A | 1598 | 38 | 0 | 0.36 | 1.35 | 0.23 | 0.75 | 0.28 | 0.04 |
| DOCK2 | 1748 | 38 | 0 | 0.54 | 1.57 | 0.79 | 1.09 | 0.33 | 0.54 |
| TNFSF13 | 1836 | 38 | 0 | 0.67 | 1.25 | 0.69 | 0.68 | 0.32 | 0.76 |
| CCL9 | 1913 | 49 | 0 | 1.23 | 1.8 | 2.19 | 1.56 | 1.55 | 2.1 |
| CCL6 | 1914 | 49 | 0 | 1.28 | 2.19 | 1.76 | 1.83 | 0.93 | 1.42 |
| CCL3 | 1915 | 40 | 0 | 0.54 | 2.36 | 1.42 | 0.83 | 0.8 | 1.04 |
| SECTM1B | 2094 | 37 | 0 | 1.45 | 1.77 | 1.35 | 2.67 | 1.08 | 1.73 |
| 0 (SPOT_2119) | 2119 | 12 | 0 | -0.17 | -0.76 | -0.12 | -4.25 | -0.79 | -0.07 |
| 0 (SPOT_2132) | 2132 | 3 | 0 | -0.49 | -0.94 | -0.69 | -0.58 | -1.08 | -0.56 |
| ALCAM | 2163 | 4 | 0 | -0.62 | -1.18 | -0.47 | -0.41 | -0.36 | -0.33 |
| PDIA5 | 2187 | 38 | 0 | 0.38 | 1.11 | 0.05 | 0.81 | -0.03 | 0.28 |
| EHHADH | 2214 | 1 | 0 | -0.34 | -0.91 | 0.11 | -0.01 | 0.22 | -0.52 |
| SAMSN1 | 2242 | 38 | 0 | 0.55 | 2.21 | 0.85 | 0.86 | -0.08 | 0.77 |
| MX1 | 2282 | 38 | 0 | 0.64 | 1.28 | 0.4 | 0.72 | 0.1 | 0.22 |
| CD200R1 | 2306 | 38 | 0 | 0.64 | 2.07 | 0.89 | 1.1 | 0.23 | 0.84 |
| HCLS1 | 2316 | 38 | 0 | 0.84 | 1.72 | 0.76 | 1.14 | 0.33 | 0.72 |
| PARP9 | 2321 | 38 | 0 | 0.33 | 1.29 | 0.61 | 0.59 | 0.07 | 0.27 |
| SH2B2 | 2452 | 40 | 0 | 0.44 | 1.4 | 0.72 | 0.54 | 0.42 | 0.54 |
| TRPV4\|TRPV1 | 2515 | 38 | 0 | 0.27 | 1.28 | 0.51 | 0.38 | 0.14 | 0.26 |
| OAS1K | 2641 | 31 | 0 | 0.47 | 2.35 | 0.94 | 0.56 | 0.42 | 0.14 |
| OASL | 2658 | 41 | 0 | 0.38 | 1.55 | 1.06 | 0.44 | 0.14 | 0.12 |
| OASL2 | 2659 | 41 | 0 | 0.49 | 1.04 | 1.05 | 0.73 | 0.18 | 0.38 |
| IL10 | 2701 | 38 | 0 | 0.38 | 1.78 | 0.12 | 0.16 | 0.05 | 0.14 |
| TNFSF18 | 2754 | 38 | 0 | 0.44 | 1.52 | 0.32 | 0.56 | 0.33 | 0.81 |
| CD55 | 2880 | 38 | 0 | 0.35 | 1.47 | 0.47 | 0.48 | 0.4 | 1.13 |
| PTPRV | 2891 | 20 | 0 | -0.8 | -1.89 | -0.94 | -0.95 | -0.63 | -0.72 |
| LOC685707 | 2894 | 49 | 0 | 0.89 | 1.06 | 0.68 | 1.1 | 0.68 | 0.77 |
| NAV1 | 2895 | 49 | 0 | 0.6 | 1 | 0.66 | 0.98 | 0.61 | 0.86 |
| RGD1311892 | 2897 | 20 | 0 | -0.24 | -1.49 | -0.32 | -0.49 | -0.37 | -0.44 |
| PTPRC | 2903 | 49 | 0 | 1.12 | 2.34 | 1.44 | 1.61 | 0.85 | 1.55 |
| PRG4 | 2919 | 49 | 0 | 1.55 | 3.03 | 2.75 | 3.05 | 2.86 | 2.78 |
| FCGR2B | 2969 | 49 | 0 | 2.03 | 2.7 | 2.5 | 2.61 | 1.25 | 1.99 |
| FCGR2A\|LOC498276\|FCGR2B | 2970;2971 | 38 | 0 | 0.91 | 1.42 | 0.85 | 1.22 | 0.27 | 0.62 |
| FCER1G | 2972 | 49 | 0 | 1.21 | 1.95 | 1.39 | 1.55 | 0.91 | 1.44 |
| HLX | 3000 | 38 | 0 | 0.52 | 1.4 | 0.46 | 0.67 | 0.23 | 0.49 |
| CXCL10 | 3044 | 41 | 0 | 0.86 | 2.07 | 1.31 | 0.86 | 0.21 | 0.29 |
| CXCL9 | 3045 | 49 | 0 | 1.38 | 2.1 | 1.84 | 0.83 | 0.66 | 1.22 |
| TMPRSS11E | 3061 | 4 | 0 | -0.63 | -1.97 | -0.67 | -0.48 | -0.05 | -0.19 |
| TLR6 | 3096 | 38 | 0 | 0.37 | 1.31 | 0.55 | 0.57 | 0.13 | 0.55 |
| LOC289641 | 3100 | 20 | 0 | -0.61 | -1.13 | -0.86 | -0.75 | -0.74 | -0.6 |
| TNIP2 | 3129 | 49 | 0 | 0.56 | 1.54 | 0.95 | 0.66 | 0.35 | 0.61 |
| OSM | 3139 | 38 | 0 | 0.23 | 1.26 | 0.29 | 0.15 | 0.07 | 0.11 |
| NIPSNAP1 | 3141 | 4 | 0 | -0.66 | -1.09 | -0.59 | -0.57 | -0.45 | -0.38 |
| 0 (SPOT_3187) | 3187 | 38 | 0 | 0.22 | 1.28 | 0.17 | 0.42 | 0.21 | 0.07 |
| ANXA3 | 3205 | 38 | 0 | 0.42 | 1.27 | 0.14 | 0.41 | -0.41 | 0.45 |
| CXCL2 | 3216 | 38 | 0 | 0.13 | 2.94 | 0.38 | 0.04 | 0.07 | 0.01 |
| CXCL1 | 3217 | 38 | 0 | 0.39 | 3.35 | 0.35 | 0.52 | 0.06 | 0.07 |
| PF4 | 3218 | 38 | 0 | 0.6 | 1.51 | 0.29 | 0.75 | 0.06 | 0.48 |
| CD38 | 3278 | 44 | 0 | 1.18 | 2.27 | 1.82 | 1.11 | -0.24 | 0.9 |
| SLC39A2 | 3396 | 22 | 0 | -0.25 | -1.17 | -0.73 | -0.29 | 0.07 | -0.28 |
| LCP1 | 3467 | 49 | 0 | 0.84 | 1.8 | 1.2 | 1.38 | 0.87 | 1.07 |
| 0 (SPOT_3498) | 3498 | 38 | 0 | 0.85 | 1.76 | 0.61 | 0.97 | -0.03 | 1.88 |
| GCH1 | 3537 | 38 | 0 | 0.21 | 1.57 | 0.49 | 0.22 | -0.06 | 0.22 |
| RGD1561955 | 3645 | 49 | 0 | 0.43 | 1.13 | 0.85 | 0.84 | 0.53 | 0.71 |
| GPR183 | 3672 | 38 | 0 | 1.06 | 1.91 | 0.5 | 1.59 | 0.65 | 1.08 |
| WNT5A | 3682 | 13 | 0 | -1.11 | -1.52 | -1.08 | -0.45 | -0.25 | -0.29 |
| CDKN2AIP | 3771 | 11 | 0 | -0.36 | -0.63 | -0.88 | -1.16 | -0.64 | -0.21 |
| ADPRHL1 | 3817 | 39 | 0 | 0.11 | 1.44 | 0.44 | 1.27 | 1.58 | 1.42 |
| RGD1564899 | 3850;3851;3852 | 18 | 0 | 0.17 | 1.47 | 0.63 | 1.1 | 1.54 | 0.92 |
| SNCG | 3860 | 2 | 0 | 0.03 | -0.5 | -0.92 | -0.33 | -0.3 | -1.16 |
| PLVAP | 3880 | 38 | 0 | 0.56 | 1.25 | 0.27 | 0.76 | 0.04 | 0.29 |
| SLC20A2 | 3951 | 4 | 0 | -0.74 | -1.33 | -0.49 | -1.08 | -0.32 | -0.36 |
| GATA3 | 4089 | 35 | 0 | -0.55 | -2.17 | -0.31 | -0.45 | -0.07 | 0.03 |
| SERPINB9 | 4180 | 38 | 0 | 0.45 | 1.5 | 0.01 | 0.7 | 0.08 | 0.07 |
| GPLD1 | 4190 | 35 | 0 | -0.26 | -1.78 | -0.28 | -0.56 | 0.03 | -0.12 |
| COLEC12 | 4267 | 38 | 0 | 0.76 | 1.06 | 0.11 | 0.86 | -0.08 | 0.54 |
| CSF1R | 4346 | 49 | 0 | 1 | 1.38 | 0.99 | 1.32 | 0.64 | 0.88 |
| AQP4 | 4399 | 10 | 0 | -1.14 | -0.46 | -1.3 | -1.94 | -1.93 | -1.8 |
| CD14 | 4429 | 41 | 0 | 1.1 | 2.42 | 2.14 | 1.62 | 0.81 | 0.73 |
| HMOX1 | 4523 | 17 | 0 | 0.4 | 2.27 | 0.57 | 0.38 | -0.02 | -0.27 |
| IRF8 | 4610 | 49 | 0 | 1.21 | 1.7 | 1.01 | 1.39 | 0.79 | 1.25 |
| CD180 | 4815 | 38 | 0 | 0.52 | 1.54 | 0.02 | 1.52 | 0.71 | 0.73 |
| C6 | 4838 | 38 | 0 | 1.54 | 2.34 | 0.7 | 0.94 | 0.45 | 1.29 |
| LOC685385 | 5001 | 20 | 0 | -0.63 | -2.45 | -0.91 | -1.23 | -0.84 | -0.64 |
| CTSS | 5033 | 49 | 0 | 1.19 | 1.67 | 1.27 | 0.95 | 0.67 | 1.2 |
| LEF1 | 5097 | 4 | 0 | -1.73 | -1.91 | -1.04 | -1.7 | -0.29 | -0.18 |
| GBP2 | 5115 | 38 | 0 | 0.67 | 1.45 | 0.35 | 0.91 | 0.2 | 0.28 |
| MEF2C | 5141 | 48 | 0 | 0.46 | 1.03 | 0.52 | 1.07 | 1.37 | 0.75 |
| IL7R | 5201 | 40 | 0 | 0.6 | 2.01 | 1.43 | 0.71 | 0.44 | 0.75 |
| TLR2 | 5308 | 38 | 0 | 0.51 | 2.32 | 0.54 | 0.76 | 0.17 | 0.35 |
| FCGR1A | 5372 | 49 | 0 | 1.29 | 2.19 | 1.52 | 2 | 0.83 | 1.42 |
| CSF1 | 5403 | 49 | 0 | 0.65 | 1.08 | 0.76 | 1.07 | 0.06 | 0.71 |
| TNF | 5515 | 49 | 0 | 0.55 | 1.03 | 0.66 | 0.53 | 0.22 | 0.4 |
| LST1 | 5516 | 49 | 0 | 0.38 | 1.21 | 0.79 | 0.8 | 0.3 | 0.39 |
| AIF1 | 5517 | 49 | 0 | 0.79 | 1.56 | 0.83 | 1.32 | 0.41 | 0.85 |
| C2\|CFB | 5521 | 49 | 0 | 1.47 | 2.04 | 1.69 | 1.66 | 0.82 | 1.37 |
| C4-2\|C4B | 5522 | 49 | 0 | 1.06 | 1.46 | 1.08 | 1.06 | 0.74 | 1.27 |
| PSMB9 | 5529 | 38 | 0 | 0.56 | 1.15 | 0.17 | 0.89 | 0.06 | 0.14 |
