# Supplementary material for: Identification of hub genes related to silicone-induced immune response in rats
Source: Oncotarget. 2017 Oct 6;8(59):99772–83. doi: 10.18632/oncotarget.21546 (PMC5725130; doi:10.18632/oncotarget.21546)
Supplement: Supplementary file 3 [file oncotarget-08-99772-s003.docx]

**Supplementary Table 2: Expression information of two statistically significant profiles (80 genes) in terms of both expression pattern and GO term enrichment.**

**Profile 38**

| genesymbol | spot_id | 0 | 7 | 8 | 9 | 10 | 11 | 14 |
| --- | --- | --- | --- | --- | --- | --- | --- | --- |
| ENPP3 | 35 | 0 | 0.72 | 1.58 | 0.46 | 1.34 | 0.18 | 0.7 |
| TGFB1 | 169 | 0 | 0.67 | 1.17 | 0.47 | 0.9 | 0.43 | 0.75 |
| ITGAL | 422 | 0 | 0.63 | 1.54 | 0.51 | 1.28 | 0.48 | 0.64 |
| JAK2 | 580 | 0 | 0.74 | 2.05 | 0.93 | 0.82 | 0.24 | 0.59 |
| CD274 | 581 | 0 | 0.53 | 1.86 | 0.56 | 0.57 | 0.38 | 0.6 |
| NFKB2 | 625 | 0 | 0.39 | 1.44 | 0.73 | 0.77 | 0.37 | 0.3 |
| STX11 | 667 | 0 | 0.3 | 2.02 | 0.69 | 0.56 | 0.25 | 0.36 |
| FCGRT | 884 | 0 | 0.85 | 1.16 | 0.58 | 0.62 | 0.25 | 0.75 |
| FES | 958 | 0 | 0.61 | 1.46 | 0.83 | 0.75 | 0.21 | 0.48 |
| IFIT1\|IFIT1LB | 1249 | 0 | 0.44 | 3.38 | 0.72 | 1.11 | 0.13 | 0.25 |
| PIK3AP1 | 1269 | 0 | 0.32 | 1.26 | 0.57 | 0.8 | 0.23 | 0.52 |
| ADORA2B | 1427 | 0 | 0.94 | 2.95 | 0.75 | 0.81 | 0.05 | 0.4 |
| NOS2 | 1511 | 0 | 0.56 | 2.64 | 0.47 | 0.53 | 0.05 | 0.2 |
| CCL7 | 1524 | 0 | 1.89 | 4.19 | 1.56 | 1.74 | 0.7 | 0.87 |
| CCL4 | 1534 | 0 | 0.13 | 2.19 | 0.46 | 0.36 | 0.09 | 0.35 |
| CSF3 | 1582 | 0 | 0.08 | 1.41 | 0.08 | 0.1 | 0.09 | 0.08 |
| STAT5A | 1598 | 0 | 0.36 | 1.35 | 0.23 | 0.75 | 0.28 | 0.04 |
| DOCK2 | 1748 | 0 | 0.54 | 1.57 | 0.79 | 1.09 | 0.33 | 0.54 |
| TNFSF13 | 1836 | 0 | 0.67 | 1.25 | 0.69 | 0.68 | 0.32 | 0.76 |
| PDIA5 | 2187 | 0 | 0.38 | 1.11 | 0.05 | 0.81 | -0.03 | 0.28 |
| SAMSN1 | 2242 | 0 | 0.55 | 2.21 | 0.85 | 0.86 | -0.08 | 0.77 |
| MX1 | 2282 | 0 | 0.64 | 1.28 | 0.4 | 0.72 | 0.1 | 0.22 |
| CD200R1 | 2306 | 0 | 0.64 | 2.07 | 0.89 | 1.1 | 0.23 | 0.84 |
| HCLS1 | 2316 | 0 | 0.84 | 1.72 | 0.76 | 1.14 | 0.33 | 0.72 |
| PARP9 | 2321 | 0 | 0.33 | 1.29 | 0.61 | 0.59 | 0.07 | 0.27 |
| TRPV4\|TRPV1 | 2515 | 0 | 0.27 | 1.28 | 0.51 | 0.38 | 0.14 | 0.26 |
| IL10 | 2701 | 0 | 0.38 | 1.78 | 0.12 | 0.16 | 0.05 | 0.14 |
| TNFSF18 | 2754 | 0 | 0.44 | 1.52 | 0.32 | 0.56 | 0.33 | 0.81 |
| CD55 | 2880 | 0 | 0.35 | 1.47 | 0.47 | 0.48 | 0.4 | 1.13 |
| FCGR2A | 2970;2971 | 0 | 0.91 | 1.42 | 0.85 | 1.22 | 0.27 | 0.62 |
| HLX | 3000 | 0 | 0.52 | 1.4 | 0.46 | 0.67 | 0.23 | 0.49 |
| TLR6 | 3096 | 0 | 0.37 | 1.31 | 0.55 | 0.57 | 0.13 | 0.55 |
| OSM | 3139 | 0 | 0.23 | 1.26 | 0.29 | 0.15 | 0.07 | 0.11 |
| 0 (SPOT_3187) | 3187 | 0 | 0.22 | 1.28 | 0.17 | 0.42 | 0.21 | 0.07 |
| ANXA3 | 3205 | 0 | 0.42 | 1.27 | 0.14 | 0.41 | -0.41 | 0.45 |
| CXCL2 | 3216 | 0 | 0.13 | 2.94 | 0.38 | 0.04 | 0.07 | 0.01 |
| CXCL1 | 3217 | 0 | 0.39 | 3.35 | 0.35 | 0.52 | 0.06 | 0.07 |
| PF4 | 3218 | 0 | 0.6 | 1.51 | 0.29 | 0.75 | 0.06 | 0.48 |
| 0 (SPOT_3498) | 3498 | 0 | 0.85 | 1.76 | 0.61 | 0.97 | -0.03 | 1.88 |
| GCH1 | 3537 | 0 | 0.21 | 1.57 | 0.49 | 0.22 | -0.06 | 0.22 |
| GPR183 | 3672 | 0 | 1.06 | 1.91 | 0.5 | 1.59 | 0.65 | 1.08 |
| PLVAP | 3880 | 0 | 0.56 | 1.25 | 0.27 | 0.76 | 0.04 | 0.29 |
| SERPINB9 | 4180 | 0 | 0.45 | 1.5 | 0.01 | 0.7 | 0.08 | 0.07 |
| COLEC12 | 4267 | 0 | 0.76 | 1.06 | 0.11 | 0.86 | -0.08 | 0.54 |
| CD180 | 4815 | 0 | 0.52 | 1.54 | 0.02 | 1.52 | 0.71 | 0.73 |
| C6 | 4838 | 0 | 1.54 | 2.34 | 0.7 | 0.94 | 0.45 | 1.29 |
| GBP2 | 5115 | 0 | 0.67 | 1.45 | 0.35 | 0.91 | 0.2 | 0.28 |
| TLR2 | 5308 | 0 | 0.51 | 2.32 | 0.54 | 0.76 | 0.17 | 0.35 |
| PSMB9 | 5529 | 0 | 0.56 | 1.15 | 0.17 | 0.89 | 0.06 | 0.14 |

**Profile 49**

| genesymbol | spot_id | 0 | 7 | 8 | 9 | 10 | 11 | 14 |
| --- | --- | --- | --- | --- | --- | --- | --- | --- |
| TYROBP | 193 | 0 | 1.1 | 1.92 | 1.57 | 1.51 | 0.77 | 1.39 |
| GAB2 | 316 | 0 | 0.78 | 1.36 | 0.89 | 1.01 | 0.75 | 0.96 |
| IFITM1 | 464 | 0 | 1.72 | 2.25 | 1.79 | 2.24 | 1.15 | 1.96 |
| UNC93B1 | 484 | 0 | 0.98 | 1.6 | 1.28 | 1.59 | 0.64 | 1.41 |
| LPXN | 544 | 0 | 0.55 | 1.4 | 0.77 | 1.11 | 0.34 | 0.8 |
| IL33 | 583 | 0 | 0.9 | 2.03 | 1.3 | 1.3 | 0.1 | 1.3 |
| CD37 | 888 | 0 | 0.72 | 1.77 | 1.18 | 1.43 | 0.79 | 1.31 |
| SPN | 1093 | 0 | 0.56 | 0.91 | 0.95 | 0.92 | 0.64 | 1.24 |
| CCL2 | 1523 | 0 | 2.38 | 3.43 | 2.42 | 2.09 | 1.68 | 1.6 |
| CCL9 | 1913 | 0 | 1.23 | 1.8 | 2.19 | 1.56 | 1.55 | 2.1 |
| CCL6 | 1914 | 0 | 1.28 | 2.19 | 1.76 | 1.83 | 0.93 | 1.42 |
| LOC685707 | 2894 | 0 | 0.89 | 1.06 | 0.68 | 1.1 | 0.68 | 0.77 |
| NAV1 | 2895 | 0 | 0.6 | 1 | 0.66 | 0.98 | 0.61 | 0.86 |
| PTPRC | 2903 | 0 | 1.12 | 2.34 | 1.44 | 1.61 | 0.85 | 1.55 |
| PRG4 | 2919 | 0 | 1.55 | 3.03 | 2.75 | 3.05 | 2.86 | 2.78 |
| FCGR2B | 2969 | 0 | 2.03 | 2.7 | 2.5 | 2.61 | 1.25 | 1.99 |
| FCER1G | 2972 | 0 | 1.21 | 1.95 | 1.39 | 1.55 | 0.91 | 1.44 |
| CXCL9 | 3045 | 0 | 1.38 | 2.1 | 1.84 | 0.83 | 0.66 | 1.22 |
| TNIP2 | 3129 | 0 | 0.56 | 1.54 | 0.95 | 0.66 | 0.35 | 0.61 |
| LCP1 | 3467 | 0 | 0.84 | 1.8 | 1.2 | 1.38 | 0.87 | 1.07 |
| RGD1561955 | 3645 | 0 | 0.43 | 1.13 | 0.85 | 0.84 | 0.53 | 0.71 |
| CSF1R | 4346 | 0 | 1 | 1.38 | 0.99 | 1.32 | 0.64 | 0.88 |
| IRF8 | 4610 | 0 | 1.21 | 1.7 | 1.01 | 1.39 | 0.79 | 1.25 |
| CTSS | 5033 | 0 | 1.19 | 1.67 | 1.27 | 0.95 | 0.67 | 1.2 |
| FCGR1A | 5372 | 0 | 1.29 | 2.19 | 1.52 | 2 | 0.83 | 1.42 |
| CSF1 | 5403 | 0 | 0.65 | 1.08 | 0.76 | 1.07 | 0.06 | 0.71 |
| TNF | 5515 | 0 | 0.55 | 1.03 | 0.66 | 0.53 | 0.22 | 0.4 |
| LST1 | 5516 | 0 | 0.38 | 1.21 | 0.79 | 0.8 | 0.3 | 0.39 |
| AIF1 | 5517 | 0 | 0.79 | 1.56 | 0.83 | 1.32 | 0.41 | 0.85 |
| C2\|CFB | 5521 | 0 | 1.47 | 2.04 | 1.69 | 1.66 | 0.82 | 1.37 |
| C4-2\|C4B | 5522 | 0 | 1.06 | 1.46 | 1.08 | 1.06 | 0.74 | 1.27 |
